# Bi-Functional Paraffin@Polyaniline/TiO_2_/PCN-222(Fe) Microcapsules for Solar Thermal Energy Storage and CO_2_ Photoreduction

**DOI:** 10.3390/nano12010002

**Published:** 2021-12-21

**Authors:** Wenchang Sun, Yueming Hou, Xu Zhang

**Affiliations:** Hebei key Laboratory of Functional Polymers, School of Chemical Engineering and Technology, Hebei University of Technology, Tianjin 300130, China; 201811501015@stu.hebut.edu.cn (W.S.); 201921501013@stu.hebut.edu.cn (Y.H.)

**Keywords:** microencapsulated phase change material, solar thermal energy storage, Pickering emulsion polymerization, CO_2_ photoreduction

## Abstract

A novel type of bi-functional microencapsulated phase change material (MEPCM) microcapsules with thermal energy storage (TES) and carbon dioxide (CO_2_) photoreduction was designed and fabricated. The polyaniline (PANI)/titanium dioxide (TiO_2_)/PCN-222(Fe) hybrid shell encloses phase change material (PCM) paraffin by the facile and environment-friendly Pickering emulsion polymerization, in which TiO_2_ and PCN-222(Fe) nanoparticles (NPs) were used as Pickering stabilizer. Furthermore, a ternary heterojunction of PANI/(TiO_2_)/PCN-222(Fe) was constructed due to the tight contact of the three components on the hybrid shell. The results indicate that the maximum enthalpy of MEPCMs is 174.7 J·g^−1^ with encapsulation efficiency of 77.2%, and the thermal properties, chemical composition, and morphological structure were well maintained after 500 high–low temperature cycles test. Besides, the MEPCM was employed to reduce CO_2_ into carbon monoxide (CO) and methane (CH_4_) under natural light irradiation. The CO evolution rate reached up to 45.16 μmol g^−1^ h^−1^ because of the suitable band gap and efficient charge migration efficiency, which is 5.4, 11, and 62 times higher than pure PCN-222(Fe), PANI, and TiO_2_, respectively. Moreover, the CO evolution rate decayed inapparently after five CO_2_ photoreduction cycles. The as-prepared bi-functional MEPCM as the temperature regulating building materials and air purification medium will stimulate a potential application.

## 1. Introduction

Energy and environmental issues have become two crucial obstacles restricting the construction of low-carbon energy systems [1]. Thermal energy storage (TES) is a clean and effective technology to solve the environmental crisis and reduce consumption of fossil fuels [2]. Phase change materials (PCMs), such as organics (paraffin, fatty acids, esters, alcohols, and glycols), inorganics (salt hydrates or metallic), and eutectics (mixtures of inorganic and organic), act a pivotal part in the sustainable progress of economic development under the background of increasing energy consumption due to the valuable heat storage capability, physical-chemistry durability, and small volume change [3,4]. PCMs can store heat energy at high temperatures and then release heat energy at low-temperature conditions, which shows excellent application potential in thermal insulation materials, thermo-regulated textiles, photothermal conversion, etc. [5,6,7]. However, the leakage and poor thermal conductivity of PCMs during the high–low temperature change limits the direct application under practical conditions [8]. Therefore, many encapsulated technologies (microcapsules, porous materials adsorption, blending, etc.) for confining PCMs have been developed to overcome the leakage [9,10,11].

Microencapsulation is one of the main methods of fabricating core-shell structures, where the PCMs as the core materials are confined by the shell material [12]. Generally, monolayer polymeric or inorganic shell materials were used to fabricate microencapsulated PCMs (MEPCMs). However, there are some defects that exist in the microencapsulated PCMs with polymeric shells, such as flammable properties, inferior thermos-stability, and defective thermal conductivity. In addition, the MEPCMs with inorganic shells also possess shortages of poor mechanical properties and low encapsulation efficiency [13]. Satisfactorily, MEPCMs with organic–inorganic hybrid shells integrate the superiorities and avoid the shortcomings of a single kind of shell to the most extent [14,15,16]. Nowadays, various technologies have been employed to fabricate MEPCMs with organic–inorganic hybrid shells; Pickering emulsion polymerization has been a trend to prepare MEPCMs because of its facile, nontoxic, and environmentally-friendly features, in which there is no need to add molecular emulsifiers and the pollution to the environment is also avoided [17].

Among the various prepared MEPCMs microcapsules, the multi-functional MEPCMs have aroused an increasing research interest [18,19]. The multi-functionality of MEPCMs can be achieved in the following three ways: (1) embedding nanoparticles (NPs) into the shell, (2) utilizing function-specific shell materials, and (3) designing the shape of the microcapsule [18]. Typically, one way was employed to prepare the multi-functional MEPCMs, for instance, the functional NPs ferric oxide (Fe_2_O_3_), titanium dioxide (TiO_2_), zinc oxide (ZnO), metal–organic frameworks (MOFs), and were embedded into the polymeric shell, or the functional inorganic shells like TiO_2_, cuprous oxide (Cu_2_O), tin dioxide (SnO_2_), and cadmium sulfide (CdS) were directly used for the shell to achieve the UV shielding, fluorescent, photothermal conversion, photocatalysis, antibacterial, and thermal conduction, etc. [20,21,22,23,24,25,26,27,28]. In our previous work [29], a kind of MEPCM with TiO_2_/PDVB hybrid shell was fabricated by Pickering emulsion polymerization; nevertheless, the use efficiency of single functional TiO_2_ NPs in the MEPCM is relatively low, nonfunctional PDVB shell could not achieve a synergetic combination of NPs and shell for further improving the multi-functionality especially the photocatalysis. Thus, by selecting suitable NPs and shell materials to construct heterojunction on the microcapsule, the various components can be fully contacted and entirely integrated. Every single functional component can achieve the elevated effect or new properties that are not available from a single component.

**Scheme 1:**
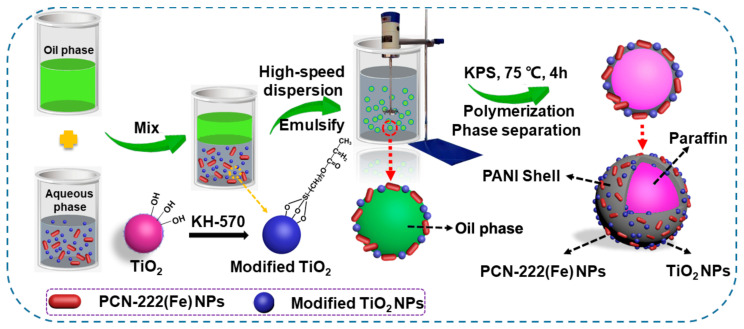
Illustration of the fabrication of paraffin@PANI/TiO_2_/PCN-222(Fe) MEPCMs by Pickering emulsion polymerization.

Herein, a novel bi-functional MEPCMs consisting of a paraffin core and a PCN-222(Fe)/titanium dioxide/polyaniline hybrid shell was prepared for solar photocatalysis and thermal storage. In the past, the polymer shell had no function other than as a shell material. Polyaniline (PANI) as the conductive polymer has attracted extensive attention because of its strong light absorption capacity and excellent photothermal conversion in a range of natural regions. However, the photocatalytic ability of PANI is restricted due to the rapid carrier recombination [30]. TiO_2_ is a kind of UV-responsive semiconductor with a high electron/hole pair complexation probability, limiting solar energy utilization for photocatalysis [31]. Moreover, numerous reports have confirmed that MOFs are excellent materials for photocatalysis, such as UiO-based MOFs, MIL-based MOFs, ZIFs-based MOFs, and Porphyrin-based MOFs [32,33,34,35,36,37,38,39] Porphyrin MOFs, such as PCN-222(Fe), are porous coordination materials formed by linking metalloporphyrins with metal carboxylate clusters [40]. However, the energy-level alignment defects and asymmetry make porphyrin MOFs have inferior photocatalytic efficiency [41]. The combination of several materials to form a heterojunction can significantly enhance the natural light absorption intensity, absorption range, and photogenerated charge migration efficiency at the interface for improving photocatalytic efficiency [42]. Therefore, a method of constructing ternary heterojunction on the surface of the organic–inorganic hybrid shell layer of MEPCMs was proposed; the detailed preparation process is shown in Scheme 1. A green Pickering emulsion polymerization was used for microencapsulating PCM with PANI/TiO_2_/PCN-222(Fe) hybrid shell, where PCN-222(Fe) and KH-570 modified TiO_2_ NPs were used as Pickering emulsion stabilizers. PCN-222(Fe) and TiO_2_ NPs were dispersed and embedded on the PANI shell after polymerization, in which the three components are contacted tightly mutually to construct a ternary heterojunction shell. The fabricated paraffin@PANI/TiO_2_/PCN-222(Fe) (PPTP) MEPCMs can not only absorb solar energy for heat storage, but the heterojunction hybrid shell can also achieve efficient separation of hole-electron pairs to improve the photocatalysis of CO_2_ into CO and CH_4_. The current work aims to develop a novel manufacturing technique for the MEPCMs and investigate their solar thermal storage capacity and photocatalytic CO_2_ reduction properties.

## 2. Results and Discussion

### 2.1. Morphology Structure of PPTP MEPCMs

**Figure 1:**
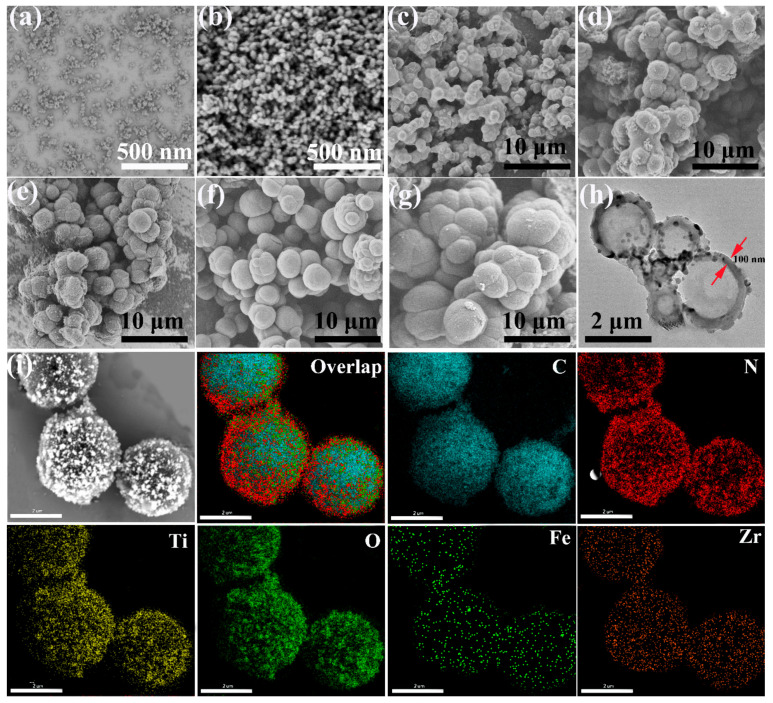
SEM images of (**a**) P25 NPs, (**b**) PCN-222(Fe) NPs, and (**c**–**g**) PPTP-1 to PPTP-5 MEPCMs. (**h**) TEM image of PPTP-3. (**i**) SEM images (CBS detector at 10 kV) of PPTP-3 and the corresponding EDX elemental mappings of overall, C, N, Ti, O, Fe, and Zr (all the plotting scales are two μm).

Figure 1a,b exhibit the SEM images of KH-570 modified TiO_2_ and PCN-222(Fe) NPs, respectively. The particle size of KH-570 modified TiO_2_ was about 30 nm. The particle size of PCN-222(Fe) NPs was around 50 nm, and their morphology was regular and generally in a fusiform shape. Figure 1c–g exhibit SEM images of PPTP-1 to PPTP-5, in which a series of MEPCMs with apparent spherical structure can be observed, where the diameter of MEPCMs increased with the decrease of the addition of TiO_2_ and PCN-222(Fe) NPs, and the MEPCMs size distribution is between 1 and 8 μm. Furthermore, the amount of TiO_2_ and PCN-222(Fe) NPs embedded on the surface of the MEPCM decreased with the continuous increase of the amount of PANI, resulting in the poor dispersion and aggregation of MEPCMs. Moreover, with the decrease of PCN-222(Fe), the size of MEPCMs accordingly decreased. Among them, PPTP-3 MEPCM has an ideal dispersion (Figure 1e), where more NPs are accumulated on the surface. The core-shell structure of PPTP-3 can be observed in the TEM image (Figure 1h), where the TiO_2_ and PCN-222(Fe) NPs embedded on the surface can also be observed. Figure 1i is the SEM image of PPTP-3 at the acceleration voltage of 10 kV, and the EDX elemental mappings of PPTP-3 show that C, N, Ti, O, Fe, and Zr elements are dispersed on the surface of MEPCMs, indicating that TiO_2_ and PCN-222(Fe) NPs are uniformly embedded on the PANI shell. The above results show that the PPTP MEPCMs with paraffin as the core and PANI/TiO_2_/PCN-222(Fe) as the hybrid shell have been successfully prepared.

### 2.2. Chemical Composition Characterization

**Figure 2:**
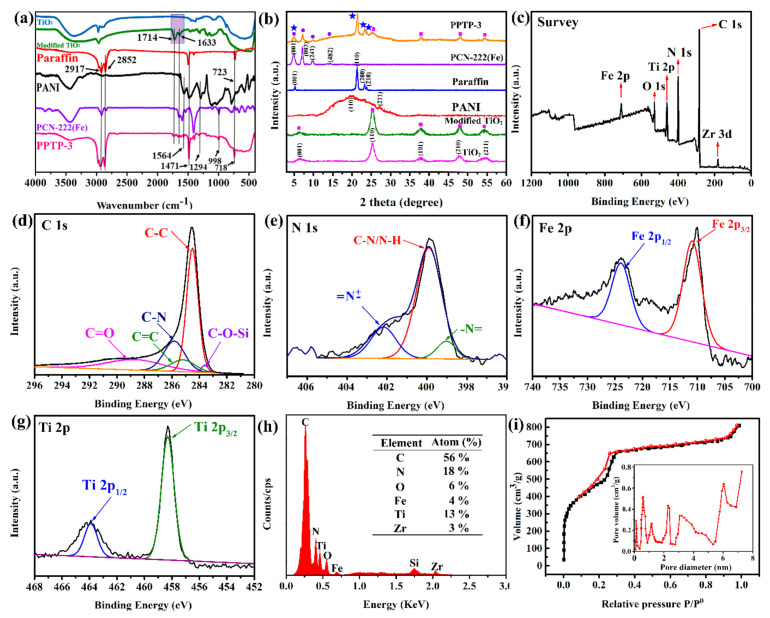
(**a**) FT-IR spectra and (**b**) XRD patterns of TiO_2_, KH-570 modified TiO_2_, paraffin, PANI, PCN-222(Fe), and PPTP-3. XPS spectra of PPTP-3: (**c**) survey scan, (**d**) C 1s, (**e**) N 1s, (**f**) Fe 2p, and (**g**) Ti 2p. (**h**) EDX spectrum and element content (inset) of PPTP-3. (**i**) N_2_ adsorption–desorption isotherms and the corresponding pore-size distributions (inset) of PPTP-3.

FT-IR spectra of TiO_2_, KH-570 modified TiO_2_, paraffin, PANI, and PPTP MEPCMs are shown in Figure 2a. For the TiO_2_, the broad peaks at around 560 and 3400 cm^−1^ are attributed to the Ti-O and hydroxyl groups stretching vibrations, respectively. The peaks of modified TiO_2_ at 1633 and 1714 cm^−1^ are attributed to C=C and C=O vibrations of KH-570, indicating the successful modification of TiO_2_ by KH-570. The peaks of paraffin at 2917 and 2852 cm^−1^ correspond to the asymmetric stretching vibration and symmetric stretching vibration of -CH_2_. The peak at 1471 cm^−1^ is attributed to -CH_2_ bending vibration, and the peak at 718 cm^−1^ is assigned to the in-plane rocking vibration of the alkyl chain. In the spectrum of PANI, the peaks at 1564 and 1471 cm^−1^ correspond to C=N and C=C stretching mode of quinone and benzene units, respectively, 1294 cm^−1^ is indexed with C-N stretching mode for benzene ring. For the spectrum of PCN-222(Fe), the symmetric Fe-N stretching at 998 cm^−1^ appears, indicating the successful complexation of Fe with porphyrin ring. In the spectrum of the PPTP-3, the characteristic peaks of the different components can be detected, suggesting that the as-prepared MEPCM consists of paraffin, TiO_2_, PANI, and PCN-222(Fe).

The crystal structures of the PPTP-3 and its various components were analyzed by XRD (Figure 2b). The crystal structures and the position of diffraction peaks hardly change after TiO_2_ modification, indicating that KH-570 grafting on the TiO_2_ surface can not affect its crystallinity. Furthermore, the characteristic diffraction peaks at 2θ of TiO_2_ are located at 25.3, 27.5, 48.1, and 54.8°, respectively. The corresponding crystal planes are (110), (101), (210), and (211), showing that TiO_2_ NPs is a crystal morphology composed of rutile and anatase mixed-phase (JCPDS No. 21-1272 and JCPDS No. 21-1276) [43]. The wide diffraction angle 2θ of PANI is around 18.4°, which is a unique characteristic diffraction peak of polymer materials [44]. Paraffin diffraction peaks at 2θ are 6.1, 21.2, 22.8, and 23.5°, which corresponds to the (001), (100), (200), and (210) crystal planes (JCPDS No. 40-1995) [45]. The diffraction peaks at 2θ = 4.8°, 7.1°, 9.8°,14.4°and 19.6° match (001), (003), (241), (161), and (482) crystal planes of PCN-222(Fe) [46]. All the diffraction peaks can be observed in the spectrum of PPTP-3, which also proves that PPTP MEPCMs are composed of paraffin, PANI, TiO_2_, and PCN-222(Fe). 

Furthermore, the elemental distribution and chemical states of PPTP-3 were characterized by XPS measurement, where the survey XPS spectra confirm the elements of C, N, Fe, O, and Ti in the PPTP-3 (Figure 2c). Four kinds of carbon species were detected in the high-resolution C 1s spectrum of PPTP-3 (Figure 2d): C=C (284.6 eV), C-N (286.1 eV), C-O-Si (283.6 eV), and C=O (288.3 eV). The high-resolution N 1s spectrum was deconvoluted into three nitrogen species (Figure 2e); the peaks at 398.1 and 399 eV are the =N- and C-N/N-H bonds of porphyrin rings in PCN-222(Fe) and PANI molecular chains, respectively. The peak at 402.4 eV is the protonated amine of PANI molecular chains [47]. The high-resolution Fe 2p spectrum was fitted into two characteristic Fe species at 710.7 (Fe 2p_3/2_) and 724.5 eV (Fe 2p_1/2_) (Figure 1f), indicating the introduction of the Fe component into the MOF nanocrystal [48]. In the high-resolution Ti 2p spectrum, signals allocated to Ti 2p_1/2_ and Ti 2p_3/2_ are observed at 463.5 and 457.9 eV (Figure 1g), which indicates the existence of Ti in a tetravalent form [49]. The XPS analysis shows the stable compound states of C, N, Fe, and Ti in the PPTP-3. In addition, the EDX spectrum of PPTP-3 is shown in Figure 2h, the contents of C, N, O, Fe, Ti, and Zr are 56, 18, 6, 4, 13, and 3 atom%, respectively, which also indicates the PPTP MEPCMs with paraffin as core and PANI/TiO_2_/PCN-222(Fe) as hybrid shell are successfully fabricated. The above results show that the MEPCMs with paraffin as the core and PANI/TiO_2_/PPTP-222(Fe) as the hybrid shell have been successfully prepared.

### 2.3. Pore Structure and Specific Surface Area Analysis

The pore structure and specific surface area of photocatalysts have an essential impact on the performance, and these parameters of materials were analyzed by the BET analyzer. The pore size of PPTP-3 was calculated according to the BET method, which was mainly concentrated at approximately 1.3, 3.2, and 3.8 nm, respectively (illustration in Figure 2i). These pore sizes match the pore sizes in PANI and PCN-222(Fe) (Figure S1), and the other larger mesopores may result from the piled pores among the MEPCM [50]. According to IUPAC classification, the N_2_ adsorption–desorption isotherm belongs to type IV, and a small amount of N_2_ adsorption appears under low relative pressure, indicating the existence of micropores. A wide H1 type hysteresis ring appears in the medium pressure region, which proves the existence of mesopores. The BET specific surface area and pore volume of PPTP-3 were 583 m^2^/g and 0.628 cm^3^/g, respectively, which well inherits the pore structure of PCN-222(Fe) and PANI.

### 2.4. Phase Change and Thermal Properties Analysis

**Figure 3:**
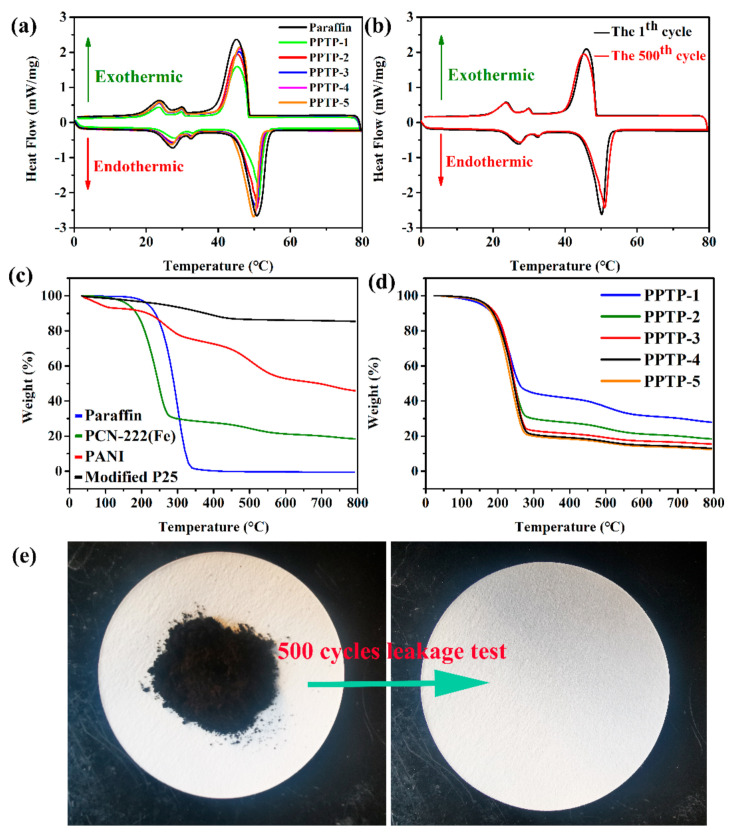
DSC curves of (**a**) paraffin and PPTP MEPCMs, and (**b**) 500 high–low temperature cycles of PPTP-3. TGA thermograms of (**c**) paraffin, PCN-222(Fe), PANI, KH-570 modified TiO_2_, and (**d**) PPTP MEPCMs. (**e**) Photos of PPTP-3 MEPCM after 500 heating-cooling cycle leaking tests.

The phase change characteristics and thermal properties of pure paraffin and PPTP MEPCMs were analyzed by DSC (Figure 3a). The curves include three solid–liquid phase change peaks in the endothermic stage and three corresponding liquid–solid phase change peaks in the exothermic stage. All the PPTP MEPCMs have similar phase change behavior compared with paraffin. Still, the melting peak temperature *T*_m_ decreases slightly, and the crystallization peak temperature *T*_c_ increases marginally due to the crystallization confinement of paraffin microencapsulated in the inner space of the hybrid shell [51]. The *T*_c_ and *T*_m_ of high melting point paraffin used in this work are 50.3 and 45.1 °C, the corresponding crystallization enthalpy Δ*H*_c_ and melting enthalpy Δ*H*_m_ are 226.3 J·g^−1^ and 228.5 J·g^−1^, respectively. A series of PPTP MEPCMs showed excellent encapsulation efficiency *E*_en_ except for PPTP-1, the *E*_en_ of PPTP-2 to PPTP-5 MEPCMs is close to 80% and had good heat storage performance (Table 1). The values of encapsulation efficiency (*E*_en_), energy storage efficiency (*E*_es_), and thermal storage capability (*C*_es_) were calculated according to Equations (S1)–(S3) in the Supporting Information. The thermal stability of PPTP-3 was verified by 500 heating–cooling cycles experiments. In Figure 3b, the enthalpy of PPTP-3 hardly decreases after 500 cycles, indicating that as-fabricated MEPCMs have superior thermal stability. Furthermore, the thermal stability of the materials was tested by TGA (Figure 3c,d). The results show that the PANI/TiO_2_/PCN-222(Fe) hybrid shell has a good protective effect on paraffin and improves its thermal stability. Significantly, the weight loss of modified P25 was attributed to the removal of KH-570 grafted on the surface of P25 during the temperature of 200~450 °C. PPTP-3 was tested for 500 high–low temperature cycles in the temperature range of 10~60 °C. As shown in Figure 3e, after the high–low temperature cycles test, no liquid paraffin remained on the filter paper, proving that PANI/TiO_2_/PCN-222(Fe) hybrid shell can prevent the internal melting core PCM from leaking.

### 2.5. Photothermal Conversion Performance

**Figure 4:**
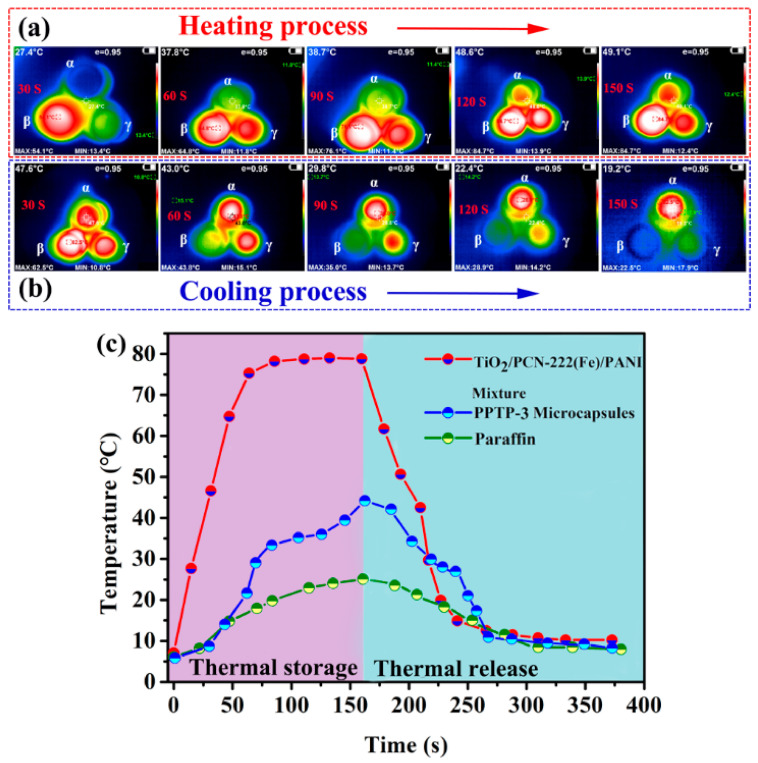
Infrared thermography images of α: paraffin, β: TiO_2_/PCN-222(Fe)/PANI mixture, and γ: PPTP-3 during the (**a**) light radiation stage and (**b**) after removing the light radiation stage. (**c**) Temperature response curves of samples over time.

To investigate the photothermal absorption-release properties of the materials, a 300 W Xenon lamp (λ > 300 nm) was used to irradiate paraffin, PCN-222(Fe)/PANI/TiO_2_ mixture (the composition ratio is the same as the shell of PPTP-3), and PPTP-3 at the same time and the surface temperature was recorded every 30 s by an infrared thermal imager (Figure 4). In the process of light irradiation and removal, it can be observed that there is an apparent difference in the surface temperature. In Figure 4a, in the course of the light irradiation, the temperature of the PCN-222(Fe)/PANI/TiO_2_ mixture continues to rise rapidly within 150 s, and the maximum temperature reaches about 85 °C. In contrast, the temperature rise speed of PPTP-3 is slightly slower than that of the mixture due to the heat absorption of internal paraffin; the maximum temperature within 150 s reaches about 49 °C. Meanwhile, the ascending speed of paraffin surface temperature is the slowest, and there is a distinct temperature hysteresis due to the poor photothermal conversion capability. In the cooling process after removing the light source (Figure 4b), in contrast to the fastest decrease of the temperature of the PCN-222(Fe)/PANI/TiO_2_ mixture, the temperature of PPTP-3 dropped slower but faster than that of paraffin. Furthermore, to observe the temperature variation, the temperature response curves of samples over time were collected (Figure 4c). During the light radiation stage, the temperature of the PCN-222(Fe)/PANI/TiO_2_ mixture almost linearly and rapidly increases to 78.5 °C in 75 s because of the ultra-high thermal conductivity of the mixture without paraffin. Nevertheless, for paraffin and PPTP-3 MEPCM, the temperatures rise rate are slow and increased to 24.8 and 44.6 °C after 160 s, respectively. After removing the irradiation light source, the temperature of the PCN-222(Fe)/PANI/TiO_2_ mixture quickly decreases from 78.5 to 12 °C within 100 s because of the high thermal conductivity of the hybrid shell. Meanwhile, the surface temperature of PPTP-3 and paraffin drops to 12 °C after cooling for respectively 120 s and 180 s due to the thermal insulation of the inner paraffin. The above results show that PCN-222(Fe)/PANI/TiO_2_ shell has superior photothermal conversion capacity than paraffin, and PPTP-3 integrates the photothermal conversion and heat energy storage, which significantly improves the thermal response and thermal regulation performance of PPTP-3.

### 2.6. Photoelectric Performance

**Figure 5:**
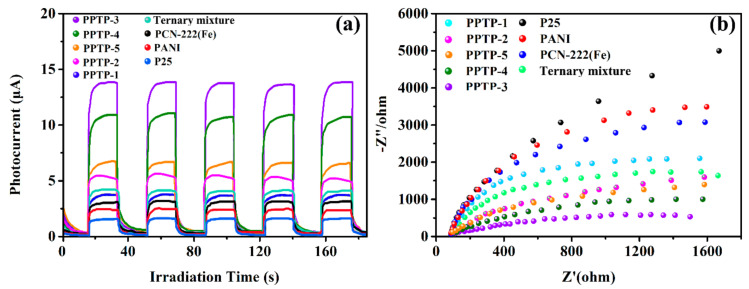
(**a**) Photocurrent-time (*I*-*t*) curves and (**b**) EIS plots of TiO_2_, PCN-222(Fe), PANI, TiO_2_/PCN-222(Fe)/PANI ternary mixture, and PPTP MEPCMs.

To investigate the photoelectrochemical characteristics of PPTP MEPCMs, TiO_2_, PCN-222(Fe), PANI, TiO_2_/PCN-222(Fe)/PANI ternary mixture, and PPTP MEPCMs were characterized by employing electrochemical workstation. The transferability of photogenerated electrons was analyzed by observing the photocurrent-time (*I*-*t*) and the EIS Nyquist plot changes. The evolution of *I*-*t* was inversely proportional to that of the EIS Nyquist plot (Figure 5). The *I*-*t* curves are shown in Figure 5a, the photocurrent intensity of the PPTP-1 to PPTP-5 MEPCMs is better than that of TiO_2_, PCN-222(Fe), PANI, and the ternary mixture. Furthermore, with the mass ratio of PCN-222(Fe)/PANI in the MEPCMs increasing first and then decreasing, the photocurrent intensity of PPTP-1 to PPTP-5 MEPCMs also increases first and then decreases, and in which the photocurrent intensity of PPTP-3 reaches the highest due to the maximum mass fraction of PCN-222(Fe) in the MEPCM. It can be observed that the EIS curves are similar to arc shape (Figure 5b), where the radian size represents the impedance value. The EIS curves show that the impedance of PPTP-3 is the smallest, indicating the optimal photoelectron–hole pair separation efficiency because of the suitable heterojunction composition ratio. In brief, the PPTP-3 ternary hybrid shell can effectively enhance the separation efficiency of electron–hole pairs in the catalyst and then improve the photocatalytic reduction capacity.

**Figure 6:**
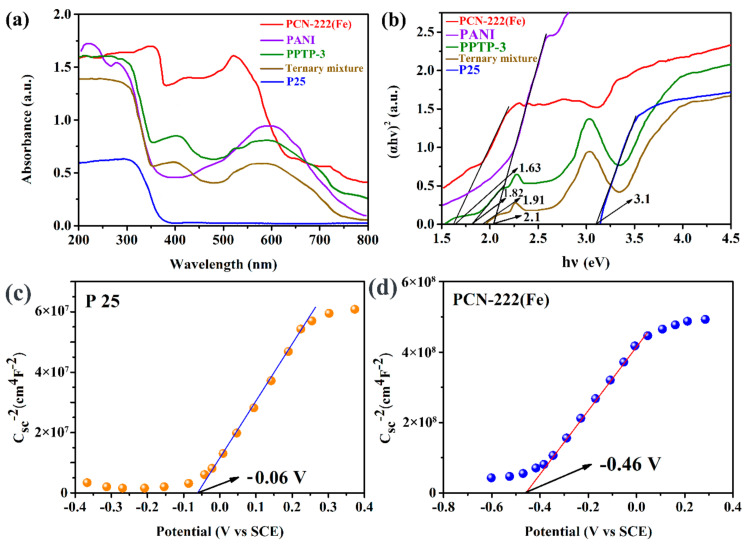
(**a**) UV-Vis diffuses reflectance spectra (UV-Vis DRS) and (**b**) band gap (*E*_g_) plots. Mott–Schottky plots of (**c**) TiO_2_ (P25) and (**d**) PCN-222(Fe).

For demonstrating the influence of heterojunction of MEPCMs shell on optical properties of composite materials, UV-Vis diffuse reflectance spectra (DRS) were further employed to investigate the light-absorbing ability (Figure 6a). Compared with pure TiO_2_ P25, pure PANI and PCN-222(Fe) have strong light absorption in the natural light region, TiO_2_/PCN-222(Fe)/PANI mixture, and PPTP-3 with ternary heterojunction have also been well inherited in light absorption ability. To clarify the effect of coupling on bandgap (*E*_g_), the *E*_g_ of various samples were calculated based on the Kubelka–Munk equation: αhv=k(hv−Eg)n/2 and (*αhv*)^n/2^ − *hv* curves (Figure 6b), where TiO_2_ is an indirect semiconductor, *n* = 4; PCN-222(Fe) and PANI are direct semiconductors, *n* = 1 [52]. Thus, the *E*_g_ of TiO_2_, PCN-222(Fe), and PANI are 3.1, 1.63, and 2.1 eV, respectively. Moreover, the *E*_g_ of the ternary mixture PCN-222(Fe)/PANI/TiO_2_ and PPTP-3 are 1.91 and 1.82 eV, respectively, indicating the integration of ternary heterojunction has more influence than the simple mixture.

To obtain the bandgap structure of each material, the flat band potentials (*E*_FB_) of TiO_2_ and PCN-222(Fe) were calculated by Mott–Schottky plots. The tangent slopes of Mott Schottky curves on TiO_2_ and PCN-222(Fe) are positive, indicating that they are n-type semiconductors (Figure 6c,d). It can be seen from the Mott–Schottky plots that the *E*_FB_ of TiO_2_ and PCN-222(Fe) are −0.06 and −0.46 V vs. Ag/AgCl, respectively. Based on Yuan’s research [53], the *E*_CB_ potential of *n*-type semiconductors is 0.2 V higher than *E*_FB_. Therefore, the *E*_CB_ values of TiO_2_ and PCN-222(Fe) are −0.26 V and −0.66 V, according to the formula *E*_g_ = *E*_VB_ − *E*_CB_; thus, the valence band potential (*E*_VB_) of TiO_2_ and PCN-222(Fe) is 2.84 V and 0.97 V, respectively. The above data provide theoretical support for the subsequent catalytic mechanism.

### 2.7. Photocatalytic Performance of Catalysts for CO_2_ Photoreduction

**Figure 7:**
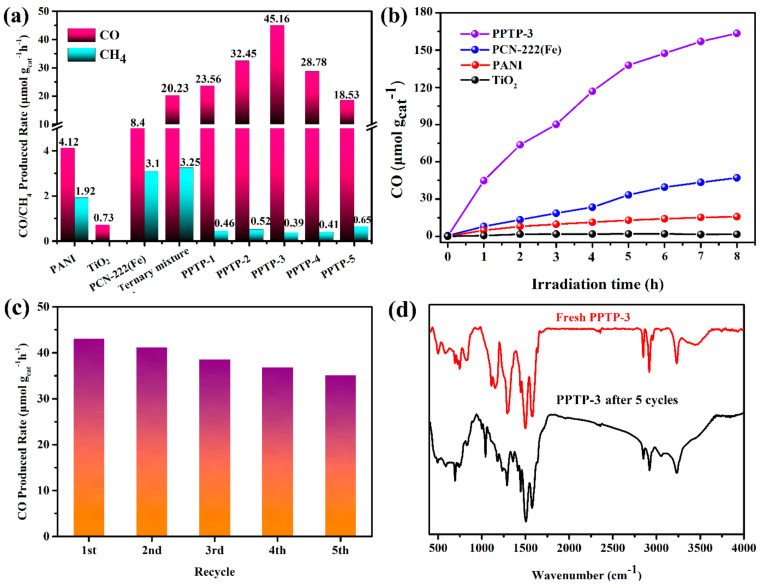
(**a**) CO and CH_4_ produced rate by PCN-222(Fe), TiO_2_, PANI, and PPTP-1 to PPTP-5 under 300 W Xe lamps. (**b**) CO reduction yield by PCN-222(Fe), TiO_2_, PANI, and PPTP-3 within 8 h. (**c**) CO produced rate after 5 times cycling utilization of PPTP-3. (**d**) FTIR spectra of PPTP-3 before and after 5 times cycling photocatalysis.

The results of CO_2_ photoreduction catalyzed by various photocatalysts are shown in Figure 7a, where the main product is CO with a small amount of CH_4_. The reduction rate of CO_2_ into CO catalyzed by PANI, TiO_2_, and PCN-222(Fe) are 4.12, 0.73, and 8.4 μmol g^−1^ h^−1^, respectively. The reduction rate of CO_2_ into CO over PPTP-1 to PPTP-5 first increases and then decreases. The PPTP-3 reaches the optimal value of 45.16 μmol g^−1^ h^−1^, which is mainly due to the highest mass fraction of PCN-222(Fe) in PPTP-3 causing the optimal photocurrent density, hole-electron separation efficiency, and suitable energy band. As the optimal photocatalyst, PPTP-3 has the highest CO yield, which is 11, 5.4, and 62 times higher than that of PANI, PCN-222(Fe), and TiO_2_, respectively. Moreover, the selectivity of CO catalyzed by PPTP MEPCMs is relatively high, where the selectivity of CO over PPTP MEPCMs is almost higher than 96%. To verify the photocatalytic specificity of heterojunction of PPTP-3, the ternary mixture (PCN-222(Fe)/PANI/TiO_2_) was also adopted to carry out the CO_2_ photoreduction, where the reduction rate of CO_2_ into CO is 20.23 μmol g^−1^ h^−1^ with the CH_4_ reduction rate of 3.25 μmol g^−1^ h^−1^, indicating the heterojunction of PPTP-3 shell has better catalytic performance because of the more favorable light absorption of the heterojunction catalyst and the weaker recombination of charges and holes between the three components. 

The results of total CO evolution yield by PCN-222(Fe), TiO_2_, PANI, and PPTP-3 with Xe lamp within 8 h are shown in Figure 7b. In the process of photoreduction within 8 h, although the reduction rate of CO_2_ catalyzed by PPTP-3 decreased slightly after 3 h, the evolution rate of CO was still maintained at a comparatively high level. The evolution amount of CO catalyzed by PPTP-3 is much higher than that of the other three single components, indicating that the ideal activity of PPTP-3 remains during the long-term catalytic process. In addition, the catalytic stability of PPTP-3 was further verified by cycle experiments (Figure 7c). After five cycles of 1 h, the CO produced rate did not decay significantly, and there was still a 90% CO produced rate compared with the first cycle. The structural stability of PPTP-3 after five cycles was verified by FTIR spectra. It can be seen from Figure 7d that the functional groups in the FTIR spectra of PPTP-3 before and after the cycles remained well.

### 2.8. Photocatalysis Mechanism of PPTP-3 MEPCM for CO_2_ Photoreduction

**Figure 8:**
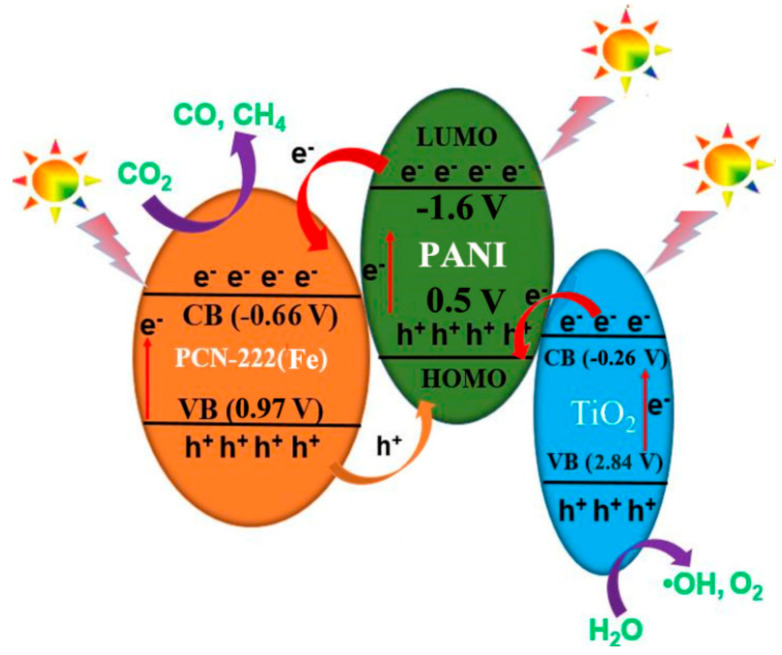
Mechanism of CO_2_ photoreduction over PPTP-3 MEPCM.

The mechanism of CO_2_ photoreduction catalyzed by PPTP-3 MEPCM is shown in Figure 8. In the hybrid shell of PPTP-3, PCN-222(Fe) and TiO_2_ NPs are dispersedly embedded on the conductive polymer PANI. PANI, as a p-type conductive polymer, can absorb natural light and further induce π-π* transition. Then, the excited state electrons transfer from the highest occupied molecular orbital (HOMO) to the lowest unoccupied molecular orbital (LUMO). The potentials of the excited state electrons on the LUMO and HOMO orbitals are 0.5 and −1.6 eV, respectively, and *E*_g_ is 2.1 eV [54], which matches the bandgap parameters of PANI in this work. Based on the above analysis of the bandgap structures of PCN-222(Fe), TiO_2_, and PANI, a ternary heterojunction of PCN-222(Fe)/TiO_2_/PANI was proposed. From the potential values of the three components, it can be seen that PCN-222(Fe) and TiO_2_ have an excellent potential match with PANI, and then an internal electric field was established. Under the irradiation of natural light, quantities of photogenerated electrons accumulated on the LUMO of PANI, and then the electrons were quickly transferred to the conduction band (CB) PCN-222(Fe). The standard potential (−0.33 V vs. NHE) of O_2_/^•^O_2_ is more negative than the CB of TiO_2_ (−0.26 eV), in which the •O_2_^−^ cannot generate in thermodynamics. Therefore, the electrons on the CB of TiO_2_ will continue to migrate to the HOMO of PANI. As such, VB of PCN-222(Fe) (0.97 eV) and HOMO potential of PANI (0.5 eV) are more negative than H_2_O/^•^OH (1.99 V vs. NHE) or OH^−^/^•^OH (2.34 V vs. NHE), which also prohibits the generation of ^•^OH. Therefore, in the process of illumination, the CB values of reduction potentials *E*^0^ (CO_2_/CO, 0.53 V vs. NHE) and *E*^0^ (CO_2_/CH_4_, 0.24 V vs. NHE) are more positive than the CB value of PCN-222(Fe) (−0.66 V vs. NHE). Most of the electrons of CB on PCN-222(Fe) were used to reduce CO_2_, to maintain the strong reducibility of CB belonging to PCN-222(Fe). MOFs tend to produce H_2_O_2_ during the photoreduction reaction. Accordingly, excessive H_2_O_2_ will consume photoinduced electrons and further weaken the reduction ability of the substrate. By compounding with other materials, such as TiO_2_ or PANI, the generation of H_2_O_2_ can be effectively reduced, thus improving the photoreduction performance. When exposed to light, photogenerated holes (H^+^) of PPTP-3 MEPCM can be produced simultaneously. Since the HOMO potential (0.5 V vs. NHE) of PANI is more negative than the VB of PCN-222(Fe), the photogenerated H^+^ on the VB of PCN-222(Fe) will transfer to the HOMO of PANI. The oxidation potential *E*^0^ (O_2_/H_2_O, 0.82 V vs. NHE) is more VB potential of TiO_2_ is more negative than the HOMO potential of PANI. Hence, H_2_O will be oxidized to O_2_ and ^•^OH on the VB of TiO_2_. In PCN-222(Fe)/TiO_2_/PANI ternary heterojunction, PANI can not only increase the concentration of photogenerated electrons on CB of PCN-222(Fe) but also absorb the photogenerated H^+^ of PCN-222(Fe) and TiO_2_, reducing the recombination of electrons and H^+^ in PCN-222(Fe) and TiO_2_. Thus, according to Equations (1)–(3) [55], the reduction of CO_2_ and oxidation of H_2_O will proceed successfully.

(1) $${\mathrm{CO}}_{2}{\;+\;2H}^{+}{\;+\;2e}^{-}\;\to {\;\mathrm{CO}\;+\;H}_{2}O\;\;\;\;\;\;\;\;\;\;\;\;\;\;\;\;\;\;\;\;E^{0}=-0.53\;V\;$$

(2) $${\mathrm{CO}}_{2}{\;+\;8H}^{+}{\;+\;8e}^{-}\;\to {\;\mathrm{CH}}_{4}{\;+\;H}_{2}O\;\;\;\;\;\;\;\;\;\;\;\;\;\;\;\;\;\;\;E^{0}=-0.24\;V\;$$

(3) $${2H}_{2}{O\;+\;4h}^{+}\;\to {\;4H}^{+}{\;+\;O}_{2}\;\;\;\;\;\;\;\;\;\;\;\;\;\;\;\;\;\;\;\;\;\;\;\;E^{0}=0.82\;V\;$$

### 2.9. Performance Comparison of MEPCMs with Organic−Inorganic Hybrid Shells in the Literature

Preparation conditions and key parameters are important references to evaluate the comprehensive performance of MEPCMs microcapsules with organic−inorganic hybrid shells. First, the emulsion process requires additional co-emulsifiers in most literature (Table S2). In this work, only the Pickering stabilizers were added without co-emulsifiers in the preparation of microcapsules, making the preparation process green and environmentally friendly. The ∆*H*_m_ and *E*_en_ of as-prepared microcapsules in this work are ideal under the condition that no co-emulsifier was added, especially ∆*H*_m_ basically achieves the highest value. The thermal stability of microcapsules was verified by 500 heating–cooling cycles, in which the enthalpy of microcapsules hardly decreases after 500 cycles. Few MEPCMs microcapsules with organic−inorganic hybrid shells have additional functions other than TES, but the integration of TES and CO_2_ photoreduction has not been seen before in the literature. Therefore, performance comparison of PPTP-3 with ternary heterojunction photocatalysts in the literature is listed in Table S3. The evolution rate of CO catalyzed by PPTP-3 exceeds most of the recently reported ternary heterojunction photocatalysts, further indicating the ternary heterojunction of the PPTP-3 shell has the superior structural advantage and light utilization efficiency.

## 3. Conclusions

In summary, we have fabricated a novel type of bi-functional paraffin@PANI/TiO_2_/PCN-222(Fe) MEPCMs with thermal energy storage and CO_2_ photoreduction ability by Pickering emulsion polymerization. The PPTP MEPCMs are composed of paraffin as PCM core and PANI/TiO_2_/PCN-222(Fe) as hybrid shell, in which the ternary heterojunction was also constructed on the shell owing to the tight contact of the three components. The MEPCMs realized the optimal phase change enthalpy of 174.7 J·g^−1^ with an encapsulation efficiency of 77.2%. The thermal properties, chemical composition, and morphological structure were retained well after 500 high–low temperature cycles test. Under natural light irradiation, the photoreduction of CO_2_ into CO and CH_4_ by the MEPCMs showed excellent activity because of the suitable band gap and enhanced separation of the photogenerated electron–hole pair. The results CO evolution rate was 45.16 μmol g^−1^ h^−1^, which is 5.4, 11, and 62 times higher than pure PCN-222(Fe), PANI, and TiO_2_, respectively. The CO evolution rate could maintain basically after five cycles of CO_2_ photoreduction within 1 h, and there was still 90% of CO yield compared with the initial test. The as-fabricated bi-functional MEPCMs will show potential applications in the energy-saving building materials and construction of photocatalytic systems due to the solar energy storage and CO_2_ photoreduction.

## Figures and Tables

**Table 1:** Thermal property date and the corresponding encapsulation parameters of various samples.

Names	Melting Process		Crystallization Process		Δ*T* (°C)	Encapsulation Parameters		
	*T*_m_ (°C)	∆*H*_m_ (J·g^−1^)	*T*_c_ (°C)	∆*H*_c_ (J·g^−1^)		*E*_en_ (%)	*E*_es_ (%)	*C*_es_ (%)
Paraffin	50.3	226.3	45.1	228.5	5.2	-	-	-
PPTP-1	52.2	138.9	45.3	139.6	6.9	61.4	61.2	99.73
PPTP-2	51.8	164.1	45.7	164.9	6.1	72.5	72.3	99.78
PPTP-3	51.4	167.7	46.2	168.8	5.2	74.1	74.0	99.85
PPTP-4	50.8	172.7	46.4	174.1	4.4	76.3	76.3	99.94
PPTP-5	50.6	174.7	46.4	176.2	4.2	77.2	77.2	99.94

## Data Availability

The data presented in this study are available on request from the corresponding author.

## Supplementary Materials

The following are available online at https://www.mdpi.com/article/10.3390/nano12010002/s1, Figure S1: N2 adsorption-desorption isotherms and the corresponding pore-size distributions (inset) of PCN-222(Fe) NPs, Table S1: Preparation recipes for PPTP MEPCMs, Table S2: Performance comparison of microencapsulated PCMs with organic-inorganic hybrid shell in the literature, Table S3: Performance comparison of ternary heterojunction photocatalysts in the literature, Equations (S1)–(S3), Experimental: Chemicals, Surface modification of TiO_2_ NPs, Ligand Synthesis Procedures, Synthesis of PCN-222(Fe) NPs, Fabrication of paraffin@PANI/TiO_2_/PCN-222(Fe) (PPTP) MEPCMs, Characterization, Electrochemical test, Photocatalytic reaction of CO_2_; Calculation formula of *E*_en_, *E*_es_, and *C*_es_. References [56,57,58,59,60,61,62,63,64,65,66,67,68,69,70,71,72,73,74,75,76,77,78,79,80] are cited in the Supplementary Materials.
