# Supplementary material for: Bi-Functional Paraffin@Polyaniline/TiO2/PCN-222(Fe) Microcapsules for Solar Thermal Energy Storage and CO2 Photoreduction
Source: Nanomaterials (Basel). 2021 Dec 21;12(1):2. doi: 10.3390/nano12010002 (PMC8746944; doi:10.3390/nano12010002)
Supplement: Supplementary file 1 [file nanomaterials-12-00002-s001.zip › nanomaterials-1460324-supplementary.pdf]

nanomaterials

Article

Bi-Functional Paraffin@Polyaniline/TiO_2/PCN-222(Fe) Microcapsules for Solar Thermal Energy Storage and CO_2 Photoreduction

Wenchang Sun, Yueming Hou and Xu Zhang *

Hebei key Laboratory of Functional Polymers, School of Chemical Engineering and Technology, Hebei University of Technology, Tianjin 300130, China; 201811501015@stu.hebut.edu.cn (W.S.); 201921501013@stu.hebut.edu.cn (Y.H.)
* Correspondence: xuzhang@hebut.edu.cn; Tel./Fax: +86-22-6020-0443

1. Experimental

1.1. Chemicals

Aniline (ANI, 98%), methacryloxy propyl trimethoxyl silane (KH-570, 98%), TiO_2 NPs (P25), paraffin (98%, melting point of 50.3 °C), ammonium persulfate (KPS), $ZrOCl_2 \cdot 8H_2O$ (98%), and hydrogen chloride (HCl, 37%) were all purchased from Tianjin Fengchuan Chemical Company. Ethanol (EtOH, 98%), N, N-dimethylformamide (DMF, 98%), and benzoic acid (BA, 98%), Iron(II) chloride tetrahydrate ($FeCl_2 \cdot 4H_2O$, 99%), anhydrous tetrahydrofuran (THF, 99.5%), propanoic acid (C_2H_5COOH, 97%), ethyl acetate (CH_3COOEt, 98%), potassium hydroxide (KOH, 85%), methyl 4-formylbenzoate (99%), pyrrole (99%), methanol (98%), chloroform ($CHCl_3$, 98%) were all purchased from Aladdin Industrial Corporation.

1.2. Surface modification of TiO_2 NPs

The surface modification of TiO_2 NPs by KH-570 is described below: 300 mL deionized H_2O was regulated pH to about four with HCl, then 4 g of TiO_2 was injected in the aqueous solution. The mixture was sonicated for 30 min, then, 3 mL of KH-570 was slowly dripped into the mixture. After that, the mixture was heated up to 80 °C with mechanically stirred for 3 h. Finally, the suspension was collected and washed with H_2O and EtOH five times, respectively. The modified TiO_2 NPs were obtained after drying at 60 °C overnight.

1.3. Ligand Synthesis Procedures.

The ligand was synthesized according to the previous report with some modifications [1,2]. The typical synthetic procedures are described as follows.

[5,10,15,20-Tetrakis(4 methoxycarbonylphenyl)porphyrin (TPPCOOMe)]

Typically, pyrrole (3.0 g, 43 mmol), methyl 4 formylbenzoate (6.9 g, 42 mmol), and propionic acid (100 mL) were added to a 500 mL three-necked flask to form a mixed solution, which was then refluxed at 140 °C for 12 h in darkness. After the reaction mixture was cooled down to room temperature, purple crystals were collected by suction filtration. Then the crystals were washed in the sequence of ethanol, ethyl acetate, and THF, and the obtained crystals were dried under vacuum at 60 °C.

[5,10,15,20-Tetrakis(4-methoxycarbonylphenyl)porphyrinato]-Fe(III) Chloride (Fe-TPPCOOMe)

Typically, TPP COOMe (0.854 g, 1.0 mmol) was dissolved in DMF solution (100 mL) containing $FeCl_2 \cdot 4H_2O$ (2.5 g, 12.8 mmol) and the mixed solution was refluxed at 160 °C for 6 h. When the mixture was cooled down to room temperature, 150 mL of H_2O was

added. The resultant precipitate was filtered and washed with 50 mL of H_2O twice. The obtained solid was dissolved in $CHCl_3$, followed by extracting three times with 1 M HCl and twice with water. The organic layer was then dried over anhydrous magnesium sulfate and evaporated to give quantitative dark brown crystals.

[5,10,15,20-Tetrakis(4 carboxyphenyl)porphyrinato] Fe(III) Chloride (Fe-TCPP)

The obtained ester (0.75 g) was stirred in THF (25 mL) and MeOH (25 mL) mixed solvent, to which a solution of KOH (2.63 g, 46.95 mmol) in H_2O (25 mL) was introduced. This mixture was refluxed for 12 h. After cooling down to room temperature, THF and MeOH were evaporated. Additional water was added to the resulting water phase and the mixture was heated until the solid was fully dissolved, then the homogeneous solution was acidified with 1M HCl until no further precipitate was detected. The brown solid was collected by filtration, washed with water, and dried in a vacuum.

1.4. Synthesis of PCN-222(Fe) NPs

The PCN-222(Fe) NPs were synthesized according to the previous report with some modifications [3]. Fe-TCPP (100 mg, 0.13 mmol, the synthesis process see the Supporting Information), 300 mg of $ZrOCl_2{\cdot}8H_2O$, and 2.2 g of BA were added into 100 mL of DMF, and then the mixture was magnetically stirred (400 rpm) at 90 °C for 4 h. Afterward, PCN-222(Fe) NPs were collected by centrifugation and then washed with DMF three times. The obtained PCN-222(Fe) NPs were dried in a vacuum oven at 120 °C overnight.

1.5. Fabrication of paraffin@PANI/TiO_2/PCN-222(Fe) (PPTP) MEPCMs

PPTP MEPCMs with TiO_2/PCN-222(Fe)/PANI ternary hybrid shell were prepared by Pickering emulsion polymerization. The formulas for PPTP MEPCMs are given in **Table S1**. First, the given mass of the TiO_2 and PCN-222(Fe) NPs were put into 120 mL of deionized H_2O and ultrasonicated for 30 min. Then, paraffin was dispersed in ANI to make up the oil phase. After the mixture of the oil phase and aqueous phase, the mixed liquor was treated with a high-speed disperser at 6000 rpm for 10 min for producing the O/W Pickering emulsion. With a stirring speed of 500 rpm, the emulsion was heated to 75 °C, and then 30 mL H_2O containing APS was injected dropwise, the reaction was maintained for 4 h. Finally, the products were collected with suction filtration and washed with H_2O and EtOH two times. The obtained PPTP MEPCMs were dried under vacuum conditions at 60 °C for 8 h.

Table S1. Preparation recipes for PPTP MEPCMs.

Entry	Sample	Paraffin (g)	ANI (mL)	TiO_2 (g)	PCN-222(Fe) (g)	APS (g)
1	PPTP-1	1.5	3	0.2	0.1	0.4
2	PPTP-2	1.5	1.5	0.2	0.1	0.2
3	PPTP-3	1.5	0.75	0.2	0.1	0.1
4	PPTP-4	1.5	0.75	0.2	0.05	0.1
5	PPTP-5	1.5	0.75	0.2	0.025	0.1

2. Characterization

Fourier transform infrared spectrometer (Bruker VECTOR-22) was used for collecting FT-IR spectra with KBr pellet method. The X-ray diffraction (XRD) patterns of the materials were collected by X-ray powder diffractometer (Bruker D8 Davinci) with Cu Kα radiation (40 kV, 150 mA) at the rate of 5° min^{-1}. X-ray photoelectron spectroscopy spectra were obtained by X-ray photoelectric spectrometer (XPS, Thermo Scientific ESCA-Lab250Xi), and the spot size was 650 μm. The binding energy was calibrated using a C 1s peak of 284.6 eV. Scanning electron microscope characterization was performed using field emission scanning electron microscopy (SEM, FEI Nova Nano-SEM450) with an acceleration voltage of 1 kV, and the surface elemental compositions were carried out by an energy-dispersive X-ray (EDX) spectrometer coupled with an acceleration voltage of 10

kV. Transmission electron microscopy (TEM) was acquired on the FEI Talos F200S. The N_2 adsorption-desorption curve and pore structure characteristics were obtained by specific surface area and porosity analyzer (ASAP 2020 M+C), and the pore size was calculated by the Brunauer-Emmett-Teller (BET) method. The thermal analysis was carried out by a differential scanning calorimeter (DSC, TA Discovery) at temperatures ranging from 0 to 80 °C with a heating/cooling rate of ± 5 °C/min^{-1} and under an N_2 atmosphere at a flow rate of 60 mL·min^{-1}. 500 melting-cooling cycle tests were carried out in a high and low-temperature test chamber (THS-250B, China) to evaluate the leakage property and cyclability. The heating range was 10 ~ 60 °C with a rate of 4 °C·min^{-1}, the cooling range was 60 ~ 10 °C with a rate of -1 °C·min^{-1}. The thermal stability of the samples was measured by thermal gravimetric analysis (TGA, SDT/Q600) under airflow with a heating rate of 10 °C·min^{-1} from 20 to 800 °C. The UV-Vis diffuse reflectance spectra (DRS) of the materials were obtained using the UV-Vis spectrophotometer (HITACHI, U-3900H) and $BaSO_4$ as the substrate. The sunlight was simulated by a 300 W xenon lamp (Irradiate the test materials at $\lambda > 300$ nm) to perform the photothermal conversion measurement. The materials were placed on the plate (10 × 10 cm^2), a thermocouple and TETO IR software were used to record the temperature change of the samples. At the same time, the xenon lamp was used to irradiate the materials, and an infrared thermal imager (ST9550) was employed to record the surface temperature every 30 s.

3. Electrochemical test

The electrochemical properties of the materials were tested by Tianjin Lanlike electrochemical workstation (LK2010). The standard three-electrode method was adopted in the test process, in which Ag/AgCl (saturated KCl) was used as the reference electrode and platinum foil (Pt) electrode was used as the counter electrode. In the test of electrochemical impedance spectroscopy (EIS), the catalyst samples suspension droplets were coated on carbon paper as working electrodes, and the electrolyte was Na_2SO_4 solution (0.5 M). In the photocurrent test, the catalyst samples suspension droplets were coated on FTO glass as working electrodes, and the electrolyte was Na_2SO_4 solution (0.5 M). Then, the electrodes were dried in an oven. During the electrochemical test, the same light source as in the reaction was used for the test. Under the bias voltage of -0.5 V, the photocurrent curves were obtained by opening/masking the light source every 20 s. The electrochemical impedance spectroscopy (EIS) curves were measured at a frequency range of 10^{-1} ~ 10^{-5} Hz, and an alternating voltage of 10 mV. Mott-Schottky curves were measured in the dark at 1000 Hz.

4. Photocatalytic reaction of CO_2

A 300 W xenon lamp ($\lambda > 300$ nm, Beijing, China, Perfect light Technology Co., Ltd.) was employed as the irradiation light source, and fixed 10 cm away from the sample. Firstly, 6 mL of deionized H_2O and 0.2 g of catalyst were injected into the quartz glass reactor, high-purity CO_2 (0.1 MPa) was filled, and vacuum treatment was carried out twice to completely remove impurities. During the reaction, the gas product was determined by a gas chromatograph (GC-7920, Beijing, China, Zhongjiao Jinyuan Technology Co., Ltd.) with flame ionization detector FID and Ar as the carrier gas. For the subsequent recycling test, the photocatalyst after the reaction was recovered by filtration, washed three times with H_2O and ethanol, and then dried in a vacuum oven for 10 h at 60 °C. The recycle test of PPTP-3 was consistent with the above test conditions, and the recycle test times were set as 5 times.

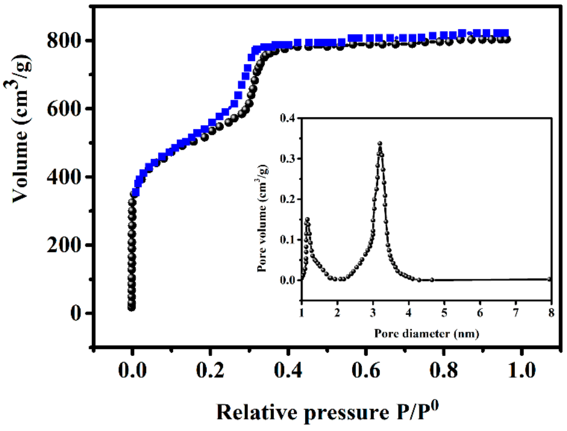

Figure S1. N_2 adsorption-desorption isotherms and the corresponding pore-size distributions (inset) of PCN-222(Fe) NPs.

Calculation formula of E_{en}, E_{es}, and C_{es}:

$$E_{en} = \frac{\Delta H_{m,\ MEPCMs}}{\Delta H_{m,\ paraffin}} \times 100\% \quad \text{Equation (S1)}$$

$$E_{es} = \frac{\Delta H_{m,\ MEPCMs} + \Delta H_{c,\ MEPCMs}}{\Delta H_{m,\ paraffin} + \Delta H_{c,\ paraffin}} \times 100\% \quad \text{Equation (S2)}$$

$$C_{es} = \frac{\Delta H_{m,\ MEPCMs} + \Delta H_{c,\ MEPCMs}}{(\Delta H_{m,\ paraffin} + \Delta H_{c,\ paraffin}) \times E_{en}} \times 100\% \quad \text{Equation (S3)}$$

Where ΔH_m, melting enthalpy; ΔH_c, crystallization enthalpy; E_{en}, encapsulation efficiency; E_{es}, energy storage efficiency; and C_{es}, thermal storage capability.

Table S2. Performance comparison of microencapsulated PCMs with organic-inorganic hybrid shell in the literature.

Sample	Preparation Methods	Co-emulsifier	ΔH_m [a] ($J \cdot g^{-1}$)	E_{en} [b] (%)	Thermal cycles times	Loss of ΔH_m ($J \cdot g^{-1}$)	Additional function	References
PCM@MgO/TiO_2/Graphite/PS	Emulsion polymerization	SDS	N	69.9	N	N	N	[4]
PCM@MF/SiO_2	Emulsion polymerization	No	167.1	N	100	Slightly	N	[5]
PCM@poly(4-MeS-co-DVB)/TiO_2	Suspension polymerization	No	174	76.6	100	Slightly	Photodegradation of methylene blue	[6]
PCM@SiO_2/TiO_2/PDA	In-situ polymerization	CTAB	125.9	N	N	N	Solar-thermal energy conversion	[7]
PCM@thermochromic pigment/PMMA	Suspension-like polymerization	TA	129.7	66.3	N	N	Termochromic property	8
PCM@poly(HDDA)/TiO_2	On-the-fly photopolymerization	No	N	N	N	N	MB Photodegradation	9
PCM@Fe_3O_4/SiO_2	Pickering emulsion polymerization and interfacial polycondensation	CTAB	169.6	71.8	100	Slightly	Magnetism	10
PCM@PMMA/PMA/Si_3N_4	Pickering emulsion polymerization	SDBS	134.6	79.9	N	N	No	11
PCM@PDA/SiO_2/Ag	Interfacial co-hydrolysis and condensation & electroless plating	CTAB	106.5	N	100	Severely	No	12
PCM@graphene/MF	In-situ polymerization	PVA and PEG-800	219.9	86.54	N	N	No	13
PCM@PU/TiO_2	Interfacial polymerization	PEG	147.7	83.3	30	Slightly	No	14
PCM/PMMA/PBA/TiO_2	Pickering emulsion polymerization	No	90.1	36.0	N	N	No	15
PCM@PMMA/TiC/SiO_2	Pickering emulsion polymerization	No	93.0	N	N	N	No	16
PCM@PDVB/SiO_2	Sol–gel and graft polymerization	CTAB	199.6	80.0	N	N	No	17
PCN-222(Fe)/PANI/TiO_2	Pickering emulsion polymerization	No	174.7	77.2	500	Slightly	CO_2 Photoreduction	This work

Note: [a] the melting enthalpy, [b] the encapsulation efficiency, N- Not mentioned.

Table S3. Performance comparison of ternary heterojunction photocatalysts in the literature.

Ternary heterojunction	Reductant	Light source	Generation rate of main products (μmol·g^{-1}·h^{-1})	References
$CdS/rGO/TiO_2$	H_2O vapor	300 W Xe lamp	CH_4: 0.12	18
$CuGaS_2$-RGO-TiO_2	Na_2S aqueous solution	300 W Xe lamp (λ > 330 nm)	CO: 0.15	19
g-C_3N_4/Pt/3DOM-TiO_2@C	H_2O vapor	300 W Xe lamp (λ ≥ 420 nm)	CO: 1.47 CH_4: 6.56	20
(Au/A-TiO_2)@g-C_3N_4	H_2O vapor	300 W Xe lamp (λ ≥ 420 nm)	CO: 21.7 CH_4: 37.4	21
WO_3-TiO_2/Cu_2ZnSnS_4	H_2O vapor	400 W Xe lamp (λ > 420 nm)	CO: 15.37 CH_4: 1.69	22
PtRu/TiO_2	H_2O vapor	300 W Xe lamp	CO: 2.6 CH_4: 38.7	23
CNT/TiO_2/Cu	H_2O vapor	300 W Xe lamp	CO: 8.1 CH_4: 1.1	24
Au/Al_2O_3/TiO_2	H_2O vapor	450 W Xe lamp	CO: 11.8	25
PCN-222(Fe)/PANI/TiO_2	H_2O vapor	300 W Xe lamp (λ > 300 nm)	CO: 45.16 CH_4: 0.39	This work

References

1. Feng, D.; Gu, Z.-Y.; Li, J.-R.; Jiang, H.-L.; Wei, Z.; Zhou, H. Zirconium-metalloporphyrin PCN-222: mesoporous metal-organic frameworks with ultrahigh stability as biomimetic catalysts. *Angew. Chem. Int. Ed.* **2012**, *51*, 10307–10310, https://doi.org/10.1002/anie.201204475.
2. Asano, N.; Uemura, S.; Kinugawa, T.; Akasaka, H.; Mizutani, T. Synthesis of biladienone and bilatrienone by coupled oxidation of tetraarylporphyrins. *J. Org. Chem.* **2007**, *72*, 5320–5326, https://doi.org/10.1021/jo070692a.
3. Park, J.; Jiang, Q.; Feng, D.; Mao, L.; Zhou, H.-C. Size-controlled synthesis of porphyrinic metal–organic framework and functionalization for targeted photodynamic therapy. *J. Am. Chem. Soc.* **2016**, *138*, 3518–3525, https://doi.org/10.1021/jacs.6b00007.
4. Sami, S.; Etesami, N. Heat transfer enhancement of microencapsulated phase change material by addition of nanoparticles for a latent heat thermal energy storage system. *Energy Rep.* **2021**, *7*, 4930–4940, https://doi.org/10.1016/j.egyr.2021.07.080.
5. Zhai, D.; He, Y.; Zhang, X.; Li, W. Preparation, Morphology, and thermal performance of microencapsulated phase change materials with a MF/SiO_2 composite shell. *Energy Fuels* **2020**, *34*, 16819–16830, https://doi.org/10.1021/acs.energyfuels.0c03121.
6. Parvate, S.; Singh, J.; Dixit, P.; Vennapusa, J.R.; Maiti, T.K.; Chattopadhyay, S. Titanium Dioxide Nanoparticle-Decorated Polymer Microcapsules Enclosing Phase Change Material for Thermal Energy Storage and Photocatalysis. *ACS Appl. Polym. Mater.* **2021**, *3*, 1866–1879, https://doi.org/10.1021/acsapm.0c01410.
7. Pornea, A.M.; Kim, H. Design and synthesis of SiO_2/TiO_2/PDA functionalized phase change microcapsules for efficient solar-driven energy storage. *Energ. Convers. Manag.* **2021**, *232*, 113801.
8. Wang, H.; Luo, J.; Yang, Y.; Zhao, L.; Song, G.; Tang, G. Fabrication and characterization of microcapsulated phase change materials with an additional function of thermochromic performance. *Sol. Energy* **2016**, *139*, 591–598, https://doi.org/10.1016/j.solener.2016.10.011.
9. Ruqaiya, A.N.; Guido, B.; Vladisavljevic, G.T. Microfluidic production of poly(1,6-hexanediol diacrylate)-based polymer microspheres and bifunctional microcapsules with embedded TiO_2 nanoparticles. *Langmuir* **2018**, *34*, 11822–11831, https://doi.org/10.1021/acs.langmuir.8b02452.
10. Jiang, F.; Wang, X.; Wu, D. Design and synthesis of magnetic microcapsules based on *n*-eicosane core and Fe_3O_4/SiO_2 hybrid shell for dual-functional phase change materials. *Appl. Energy* **2014**, *134*, 456–468, https://doi.org/10.1016/j.apenergy.2014.08.061.
11. Sun, N.; Xiao, Z. Paraffin wax-based phase change microencapsulation embedded with silicon nitride nanoparticles for thermal energy storage. *J. Mater. Sci.* **2016**, *51*, 8550–8561, https://doi.org/10.1007/s10853-016-0116-0.
12. Zhu, Y.; Chi, Y.; Liang, S.; Luo, X.; Chen, K.; Tian, C.; Wang, J.; Zhang, L. Novel metal coated nanoencapsulated phase change materials with high thermal conductivity for thermal energy storage. *Sol. Energy Mater. Sol. Cells* **2017**, *176*, 212–221, https://doi.org/10.1016/j.solmat.2017.12.006.
13. Zhao, Q.; He, F.; Zhang, Q.; Fan, J.; He, R.; Zhang, K.; Yan, H.; Yang, W. Microencapsulated phase change materials based on graphene Pickering emulsion for light-to-thermal energy conversion and management. *Sol. Energy Mater. Sol. Cells* **2019**, *203*, 110204, https://doi.org/10.1016/j.solmat.2019.110204.
14. Wang, Z.; Ma, W.; Hu, D.; Wu, L. Synthesis and characterization of microencapsulated methyl laurate with polyurethane shell materials via interfacial polymerization in Pickering emulsions. *Colloids Surfaces A: Physicochem. Eng. Asp.* **2020**, *600*, 124958, https://doi.org/10.1016/j.colsurfa.2020.124958.
15. Qiu, X.Z.; Tao, Y.; Xu, X.Q.; He, X.H.; Fu, X.Y. Synthesis and characterization of paraffin/TiO_2-P(MMA-co-BA) phase change material microcapsules for thermal energy storage. *J. Appl. Polym. Sci.* **2018**, *135*, https://doi.org/10.1002/app.46447.
16. Wang, H.; Zhao, L.; Song, G; Tang, G; Shi, X. Organic-inorganic hybrid shell microencapsulated phase change materials prepared from SiO_2/TiC-stabilized Pickering emulsion polymerization. *Sol. Energy Mater. Sol. Cells* **2018**, *175*, 102–110, https://doi.org/10.1016/j.solmat.2017.09.015.

17. Ji, X.; Chen, J.; Dang, G.; Zhang, Y.; Yang, J.; Li, W.; Zhao, Y.; Jin, Z. Preparation of phase change microcapsules with inorganic/polymer hybrid shell through a "two-step" reaction. *Polym. Sci. Ser. B* **2019**, *61*, 560–566, https://doi.org/10.1134/s1560090419050075.
18. Kuai, L.; Zhou, Y.; Tu, W.; Li, P.; Li, H.; Xu, Q.; Tang, L.; Wang, X.; Xiao, M.; Zou, Z. Rational construction of a CdS/reduced graphene oxide/TiO_2 core–shell nanostructure as an all-solid-state Z-scheme system for CO_2 photoreduction into solar fuels. *RSC Adv.* **2015**, *5*, 88409–88413, https://doi.org/10.1039/c5ra14374h.
19. Takayama, T.; Sato, K.; Fujimura, T.; Kojima, Y.; Iwase, A.; Kudo, A. Photocatalytic CO_2 reduction using water as an electron donor by a powdered Z-scheme system consisting of metal sulfide and an RGO-TiO_2 composite. *Faraday Discuss.* **2017**, *198*, 397–407, https://doi.org/10.1039/C6FD00215C.
20. Wang, C., Liu, X., He, W., Zhao, Y.; Wei, Y.; Xiong, J.; Liu, J.; Li, J.; Song, W.; Zhang, X.; et al. All-solid-state Z-scheme photocatalysts of g-C_3N_4/Pt/macroporous-(TiO_2@carbon) for selective boosting visible-light-driven conversion of CO_2 to CH_4. *J. Catal.* **2020**, *389*, 440–449, https://doi.org/10.1016/j.jcat.2020.06.026.
21. Wang, C.; Zhao, Y.; Xu, H.; Li, Y.; Wei, Y.; Liu, J.; Zhao, Z. Efficient Z-scheme photocatalysts of ultrathin g-C_3N_4-wrapped Au/TiO_2-nanocrystals for enhanced visible-light-driven conversion of CO_2 with H_2O. *Appl. Catal. B: Environ.* **2019**, *263*, 118314, https://doi.org/10.1016/j.apcatb.2019.118314.
22. Raza, A., Shen, H., Haidry, A.A.; Sun, L.; Liu, R.; Cui, S. Studies of Z-scheme WO_3-TiO_2/Cu_2ZnSnS_4 ternary nanocomposite with enhanced CO_2 photoreduction under visible light irradiation. *J. CO_2 Util.* **2020**, *37*, 260–271, https://doi.org/10.1016/j.jcou.2019.12.020.
23. Wei, Y.; Wu, X.; Zhao, Y.; Wang, L.; Zhao, Z.; Huang, X.; Liu, J.; Li, J. Efficient photocatalysts of TiO_2 nanocrystals-supported PtRu alloy nanoparticles for CO_2 reduction with H_2O: Synergistic effect of Pt-Ru. *Appl. Catal. B: Environ.* **2018**, *236*, 445–457, https://doi.org/10.1016/j.apcatb.2018.05.043.
24. Rodríguez, V.; Camarillo, R.; Martínez, F.; Jiménez, C.; Rincón, J. CO_2 photocatalytic reduction with CNT/TiO_2 based nanocomposites prepared by high-pressure technology. *J. Supercrit. Fluids* **2020**, *163*, 104876, https://doi.org/10.1016/j.supflu.2020.104876.
25. Zhao, H.; Zheng, X.; Feng, X.; Li, Y. CO_2 Reduction by plasmonic Au nanoparticle-decorated TiO_2 photocatalyst with an ultrathin Al_2O_3 interlayer. *J. Phys. Chem. C* **2018**, *122*, 18949–18956, https://doi.org/10.1021/acs.jpcc.8b04239.
